# Efficacy of coadministration of calcitonin and hyperbaric bupivacaine in spinal anesthesia for unilateral open inguinal hernia repair in tramadol abuse patients: a randomized controlled trial

**DOI:** 10.1186/s12871-026-03736-9

**Published:** 2026-03-28

**Authors:** Ibrahim Mamdouh Esmat, Ahmed Kamal Mohamed, Ihab Ahmed Gad, Noha Refaat Mohamed, Sahar Mohamed Talaat, Mohammed Sayed Shorbagy, Tarek Mohamed Ashoor

**Affiliations:** 1 Department of Anesthesia, Intensive Care and Pain Management, Faculty of Medicine, Ain-Shams University, Cairo, Egypt; 2 Department of Clinical Pathology, Faculty of Medicine, Ain-Shams University, Cairo, Egypt

**Keywords:** Postoperative pain, Tramadol abuse, Failed spinal anesthesia, Calcitonin, Intrathecal, Intravenous, Stress response, C-reactive protein

## Abstract

**Background:**

Tramadol abuse is a socio-economic problem in low resource income communities and was proposed as a cause of failed spinal anesthesia (SA). The research team evaluated coadministration of a single-shot hyperbaric bupivacaine with either intrathecal (IT) or intravenous (IV) calcitonin (CAL) for SA regarding postoperative pain control characteristics and incidence of failed SA in tramadol-abuse patients undergoing unilateral open inguinal hernia repair in a randomized controlled trial.

**Methods:**

Ninety Egyptian patients were randomized into three equal groups; IT-CAL, IV-CAL and control (C) Groups. All patients received SA, 100 IU calcitonin in IT-CAL Group, 100 IU calcitonin diluted in 10 ml IV normal saline solution (NSS) in IV-CAL Group and NSS in C-Group. The total amount of morphine (rescue analgesic) consumed 24 h following surgery was the primary outcome measure. Secondary outcomes included, incidence of failed SA, time to first request for analgesia, the duration of sensory blockade (duration of analgesia), VAS scores at rest and on movement, CRP level, patient satisfaction with postoperative (PO) pain management and side effects.

**Results:**

Out of 30 randomized patients in each group, failed SA occurred in only one patient in IT-CAL Group while occurred in 8 and 9 patients in IV-CAL and C groups, respectively (*P* = 0.018). Among the cases with successful SA, the total morphine consumption (19.1 ± 2.9 mg/24h in IT-Cal Group), serum CRP levels and area under the curve of pain scores perceived over 24 h following surgery were significantly lower in IT-Cal Group among the three groups (*P* < 0.001). Incidence of side effects was comparable between study groups (*P* > 0*.*05).

**Conclusions:**

One hundred IU intrathecal calcitonin administration as an adjuvant to hyperbaric bupivacaine was associated with lower total morphine consumption, attenuated stress response and lower incidence of failed spinal anesthesia in tramadol abuse patients undergoing unilateral open inguinal hernia repair when compared with intravenous calcitonin.

**Trial registration:**

ClinicalTrials.gov Identifier: NCT04445857**.** IRB: 6379.

**Supplementary Information:**

The online version contains supplementary material available at 10.1186/s12871-026-03736-9.

## Background

Inguinal hernia repair is one of the most frequent performed surgical procedures [1]. Spinal anesthesia (SA) is a part of many enhanced recovery after surgery (ERAS®) protocols and it is an effective method for postoperative (PO) pain relief for open inguinal herniorrhaphy in adults [1, 2]. A part from benefits of SA, failed SA or short duration of action is one of the adverse events which could happen, especially in drug abusers [3, 4]. By ruling out anatomical or technical issues in the subarachnoid area that could cause a patchy or unilateral block, the decrease in the duration of SA in the drug abusing patients could be attributed to tolerance of central and peripheral narcotic and local anesthetic receptors (both in the central and peripheral nervous system) leading to increase the dose of PO analgesics given by the anesthetist in order to relief the patient’s complaint [3, 5].

Due to its increased accessibility through prescription use for the management of both acute and chronic pain, tramadol's nonmedical use has become a significant issue globally, including in Egypt, and has increased substantially in the past several years [6]. In Egypt, tramadol abuse has spread markedly, especially among youths and the middle-aged, with many myths about its value in improving physical and sexual functions [7]. Previous studies suggested that the misuse potential of tramadol is attributed to the effects of O-desmethyltramadol on the µ-opioid receptor whereas the parent compound, tramadol, produces analgesia through inhibition of both norepinephrine and serotonin reuptake [8, 9]. Tramadol inhibits K^+^-stimulated calcitonin gene-related peptide (CGRP) release and tramadol analgesic effects are antagonized by ondansetron [10].

**Fig. 1:**
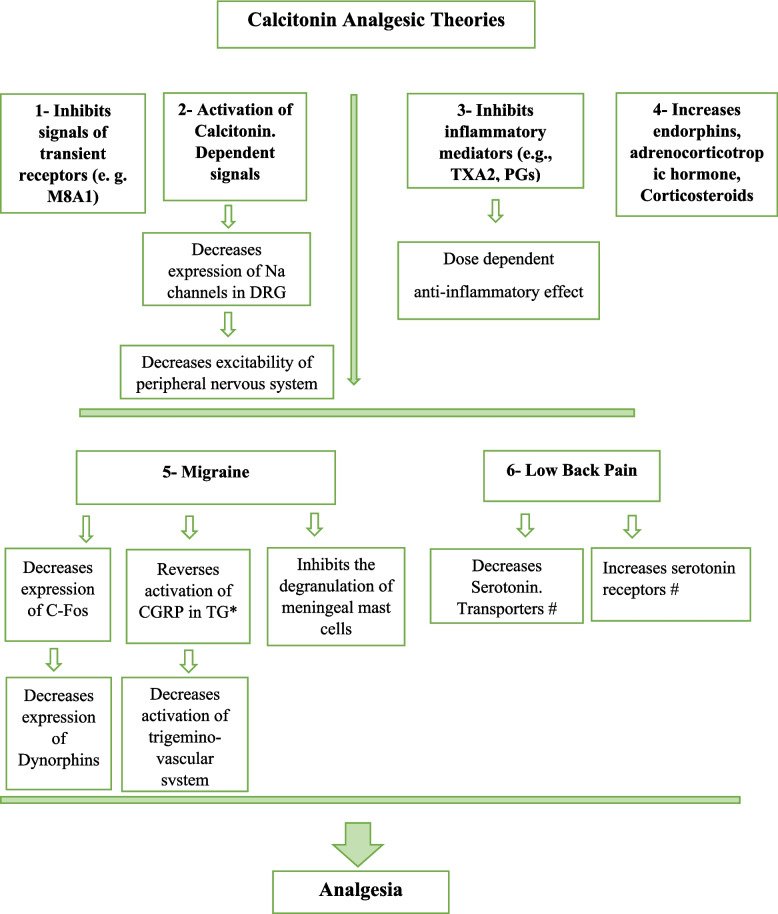
Summary of theories of Calcitonin anti-nociceptive mechanism of action (14–19). M8A1; melastatin-8 andankyrin-1, DRG; dorsal root ganglion, TXA2; thromboxane A2, PGs; prostaglandins, C-Fos; proto-oncogene, CGRP; Calcitonin gene related peptide, TG; Trigeminal ganglion. *Tramadol decreases calcitonin gene related peptide (9,10). # Tramadol inhibits both norepinephrine and serotonin reuptake (8–10)

A variety of non-narcotic adjuvants, including calcitonin, to local anesthetic that render the local anesthetic to act fast as a sensory block, to prolong its analgesic effect and to reduce the requirement of a higher dose of local anesthetic preventing the patient from potential neurotoxicity and tissue damage [11, 12]. The reduction of the enhanced expression of CGRP and c-Fos in dura, the prevention of mast cell degranulation in meninges, and the potential correlation between calcitonin and higher beta endorphin plasma levels could all be responsible for the analgesic effects of calcitonin [11]. Antinociceptive properties of calcitonin could alleviate acute pain due to a direct effect on the central serotonergic system that is independent of opioid action [13]. Figure 1 [14–19] Calcitonin exerts analgesic effects via inhibiting inflammation mediator substances, including thromboxane and prostaglandins [11, 16]. Calcitonin could be given neuro-axially (intrathecally or epidurally) or systemically (intravenous or intramuscular) for different indications to control either acute or chronic pain [11], which raise the question of the most effective route needed to achieve the best analgesic action.

Increased levels of the acute phase reactant C-reactive protein (CRP) are indicative of the stress response to surgical trauma, PO pain, and inflammation [20]. CRP was also elevated in acute low back pain (LBP), particularly in those who had high pain scores [visual analog scale (VAS ≥ 4)] [21]. Detrimental cardiometabolic consequences that can be prohibited by efficient PO analgesia, may occur in response to activation of sympathetic nervous system triggered by the stress response [22].

The research team evaluated coadministration of a single-shot hyperbaric bupivacaine with either intrathecal or intravenous calcitonin for SA regarding postoperative pain control characteristics and incidence of failed SA in tramadol-abuse patients undergoing unilateral open inguinal hernia repair.

## Materials and methods

### Ethics

On June 14, 2020, the surgery department of the Faculty of Medicine at Ain-Shams University Hospitals in Cairo, Egypt, under the direction of Chairperson Prof. Dr. Alaa Al-din Ismail, granted ethical approval for this study (IRB 6379). This study met the requirements of the 2013 Helsinki Declaration and was prospectively registered on June 24, 2020, at Clinical Trials.gov with the ID NCT04445857. Prior to the procedure, each patient provided a written informed consent, and the trial was carried out from September 1, 2020, to January 31, 2021.

### Study population

Ninety tramadol abuse Egyptian patients (single drug abuse, and not under appropriate medical supervision), regular tramadol users > 1 year, aged 18 to 60 years, and with a body mass index (BMI) < 35 kg/m^2^, who were scheduled for elective open unilateral inguinal herniorrhaphy under SA and had an American Society of Anesthesiologists (ASA) physical status II, participated in a double-blind randomized controlled study. This study included every patient with tramadol use disorder which was diagnosed when a person met specific criteria from the Diagnostic and Statistical Manual of Mental Disorder (5th ed.) (DSM-5), and was categorized as moderate [23]. The exclusion criteria included coagulopathy, neurological disease, psychological disorder, regular use of steroids, chronic use of sedatives or antidepressants, regular alcohol consumption, regular use of serotonin related medications (e.g., selective serotonin reuptake inhibitor), uncontrolled hypertension, uncontrolled diabetes mellitus, generalized or local skin infections, and duration of surgery more than 120 min. Patients with substance abuse other than tramadol, allergy to study medications, unwritten approval, renal, cardiac or hepatic failure were also excluded.

On the night before the procedure, one of the investigators performed a preoperative evaluation, a thorough explanation of the study procedures and a skin test for salmon calcitonin to all patients who had positive urine screening results for tramadol abuse. In the meanwhile, patients were advised to continue taking their regular daily dosage of tramadol to avoid experiencing an acute pain episode as a result of tramadol's opioid effects [9]. All patients were instructed for 6 h preoperative fasting for solid food and 2 h for clear fluids. Before SA, an anesthetist prepared the research medications without participating in patient administration or observation. Premedication included ranitidine 150 mg orally, and 100 mg IV hydrocortisone followed by an IV pheniramine hydrogen maleate (45.5 mg/2 ml) ampoule, one hour before SA. After surgery, ward nurses reported preoperative patient education on how to assess PO pain severity using the visual analog scale (VAS), where 0 represents no pain and 10 represents the greatest pain possible [24].

### Randomization and blinding

Based on computer-generated random numbers within sealed opaque envelopes were utilized to allocate patients into three groups (30 per group); intrathecal (IT) calcitonin (Cal) (IT-Cal Group): addition of 100 IU salmon calcitonin (1ml) to IT 15 mg (3ml) hyperbaric bupivacaine 0.5% and 10 ml IV normal saline solution (NSS) (0.9*%*) before SA; intravenous (IV) calcitonin (IV-Cal Group): addition of 1 ml NSS to IT 15 mg (3ml) hyperbaric bupivacaine 0.5% and 100 IU salmon calcitonin diluted in 10 ml IV NSS before SA, and control (C) Group: addition of 1 ml NSS to IT 15 mg (3ml) hyperbaric bupivacaine 0.5% and 10 ml IV NSS before SA. The anesthesiologists who performed the subarachnoid block and documented the sensory levels and study parameters were blinded to the patients’ group. All surgical procedures were performed by the same team of surgeons.

### Study protocol

A standardized anesthetic management was applied for all the patients. On arrival at the operating room, standard monitoring was applied including pulse oximetry, noninvasive arterial blood pressure and electrocardiography. All patients received IV lactated Ringer’s solution at 10 ml/kg 30 min before commencing SA then at 6 ml/kg/h. Following the guidelines for antiseptic technique, subarachnoid spinal block was instituted at either the L4-5 or L5-S1 interspaces (midline approach) with the patient at sitting posture using a 25 G Quincke spinal needle. After intrathecal administration of drug, the patient was positioned supine and T8 to T10 level of anesthesia was achieved using position maneuvers. All patients received supplemental oxygen via nasal cannula at a rate of 3—5 L/minute. The time to peak sensory block level (min) (time taken from the end of subarachnoid injection to loss of pin prick sensation at T8-T10 dermatome), duration of sensory blockade (duration of analgesia) (min) and complete motor blockade time (min) (time taken from the end of subarachnoid injection to the development of grade 3 motor block using the modified Bromage score [25]) and duration of motor blockade (time required for motor blockade return to Bromage grade 0 from the time of onset of motor blockade) were recorded. The duration of sensory blockade was defined as the time from the onset of successful SA to the first occasion when the patient complained of pain VAS score > 3 in the PO period. General anesthesia was given to patients who did not experience sensory block up to T10 and grade 3 motor block until 20 min after executing SA; these patients were then included in the study's final analysis. Changes produced in hemodynamic variables [heart rate (HR) & mean arterial pressure (MAP)] of patients were recorded at the following time points: baseline (pre-spinal) (Tpre), 20 min after intrathecal injection (Tpost), 2 h (T2), 4 h (T4), 6 h (T6), 12 h (T12), 24 h (T24) after surgery. The presence and severity of pain at rest (VAS-R) and on movement (VAS-M) were assessed using VAS score at the following time points: 30 min, 2,4,6,12, and 24 h postoperatively.

### Postoperative analgesia

After surgery, all patients were given 1 gm IV paracetamol every 6 h and 30 mg of ketorolac (ampoule) diluted in 100 mL of NSS were infused intravenously over 15 min every 12 h. Patients who experienced pain for the first time, postoperatively, were assessed for severity of pain at rest (VAS-R) using VAS score. If the VAS-R score was > 3, it was recorded (end point of duration of sensory blockade) (end point of duration of analgesia), morphine titration protocol was initiated [26]. Assessment of PO sedation was accomplished using a three-point sedation scale every 4 h till 24 h after the surgical procedure [(0 = alert, oriented; 1 = drowsy, oriented; 2 = drowsy, disoriented)] [27]. A bolus of 2 mg IV morphine was given and flushed with 10 ml of NSS to be followed by reassessment of pain after 5 min. This process was allowed to be repeated till VAS score decreased below 3. Also, this process was interrupted if the patient developed sedation score 2, or respiratory depression (RD) (respiratory rate < 8 breaths/min or SpO_2_ level ≤ 90%) [27]. The initial total loading morphine consumption (mg) till VAS score decreased below 3 and the total morphine consumption mg/24h were recorded.

Side effects such as hypotension (MAP is 25% less than base-line), bradycardia (HR < 50 beats/min), RD, postoperative nausea and vomiting (PONV), headache, shivering, pruritus and allergic reactions were documented. A 250 ml crystalloid bolus infusion and/or 10 mg IV ephedrine bolus doses were used to treat hypotension. 0.5 mg IV atropine was used to treat bradycardia. Patients who experienced nausea that persisted for more than ten minutes or vomiting without hypotension were given 10 mg IV metoclopramide as a rescue antiemetic. Satisfaction of all patients was surveyed 24 h after surgery using this scale (dissatisfied, neutral, or satisfied) regarding the PO analgesia regimen.

The primary outcome of this study was the total morphine consumption 24 h after surgery. Secondary outcomes included, incidence of failed SA, time to first request for analgesia, the duration of sensory blockade (duration of analgesia), VAS scores at rest and on movement (R/M), CRP level, patient satisfaction with PO pain management and side effects.

#### Laboratory analysis

Urine screening and Urine samples

Fresh urine samples were collected in sterile well capped cups for patients at anesthesia clinics, a week before surgery, without earlier announcement to avoid substitution of drug-free specimens under supervision of trustful personnel. Urine Samples were tested using Rapid Drugs of Abuse (7 tests) and positive results were confirmed by chromatographic method (Supplement 1). Positive results indicated only the presence of a drug/metabolite and did not indicate or measure intoxication. The clinical pathology specialist obtained another sample for testing if adulteration of the sample was suspected.

Blood samples

A sample of venous blood (2 mL each) was withdrawn under complete aseptic conditions from each study participant one hour (H0) preoperatively and 24 h (H1) postoperatively for assessment of CRP (Supplement 1).

### Statistical analysis

#### Power of the study

Based on findings from an earlier pilot study that our research team carried out, which demonstrated that the total morphine consumption (mg/24h) in IT-Cal, IV-Cal and C groups were 21.2 ± 2.3, 24.8 ± 4.1 and 25.6 ± 3.6 min respectively, based on the PASS 11th release program for sample size calculation [28], a minimum sample size of 20 cases per group was needed to obtain a statistically significant difference between IT-Cal and each of the IV-Cal and C groups, with power set at 80% and alpha error at 0.017 for comparisons between the three groups [29]. In order to account for failed SA cases and sample attrition, the sample size was increased to 30 patients per group.

### Data analysis

IBM SPSS statistics (Statistical Package for Social Sciences) software version 28.0, IBM Corp., Chicago, USA, 2021, was used to code, tabulate, and statistically analyze the gathered data. Quantitative data were tested for normality using Shapiro–Wilk test, then were described as mean ± standard deviation (SD) as well as minimum and maximum of the range, and then were compared using, ANOVA test, RMANOVA test and Paired t-test. Qualitative data were described as number and percentage and then were compared using Chi square test and Fisher’s Exact test. Log rank test was used to compare rate of first request for rescue analgesia. Bonferroni and Dunnett’s tests were applied for post hoc comparisons. When the p-value was less than 0.050, it was considered significant; otherwise, it was considered non-significant.

## Results

**Fig. 2:**
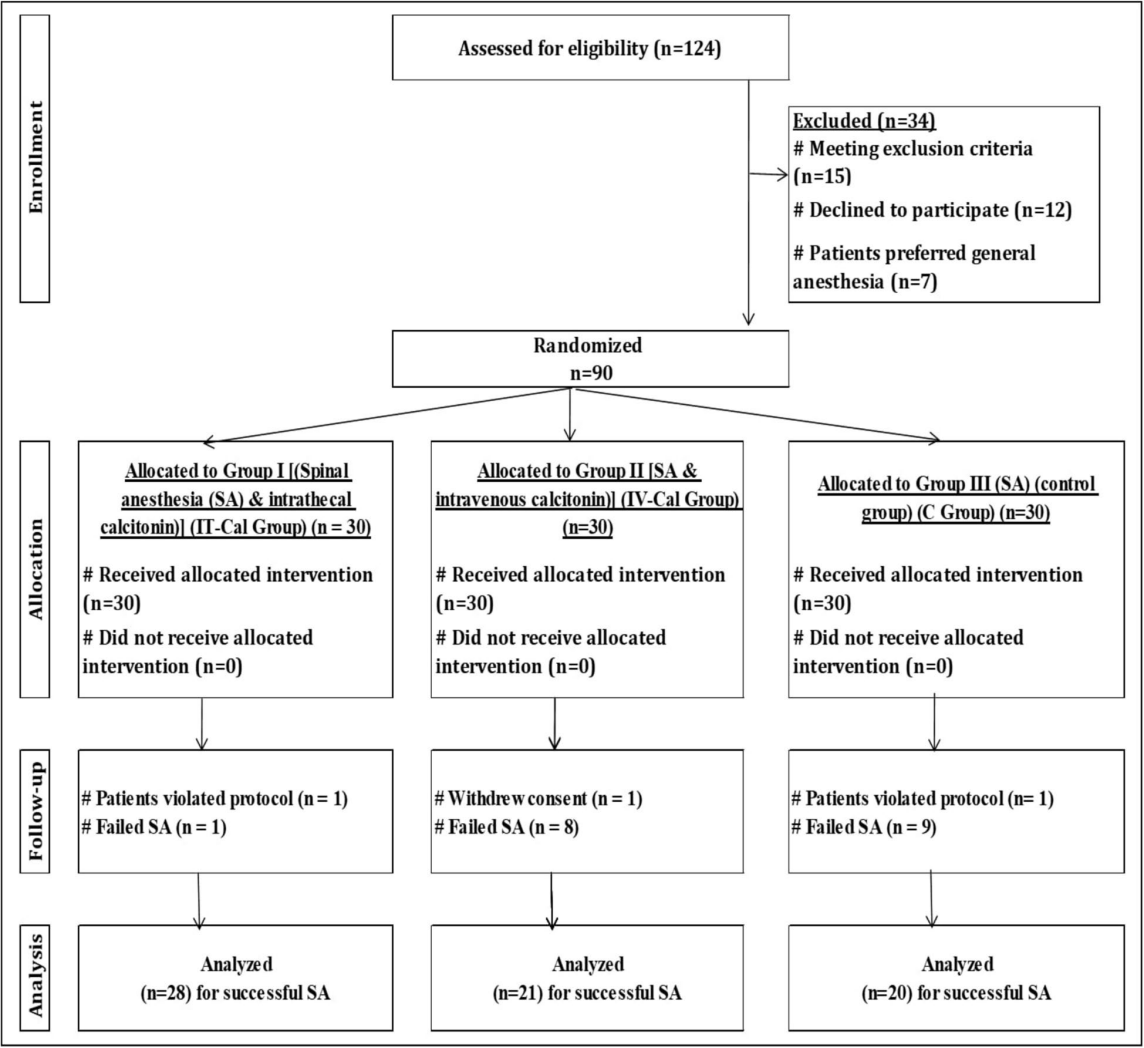
Flow diagram of the study

**Fig. 3:**
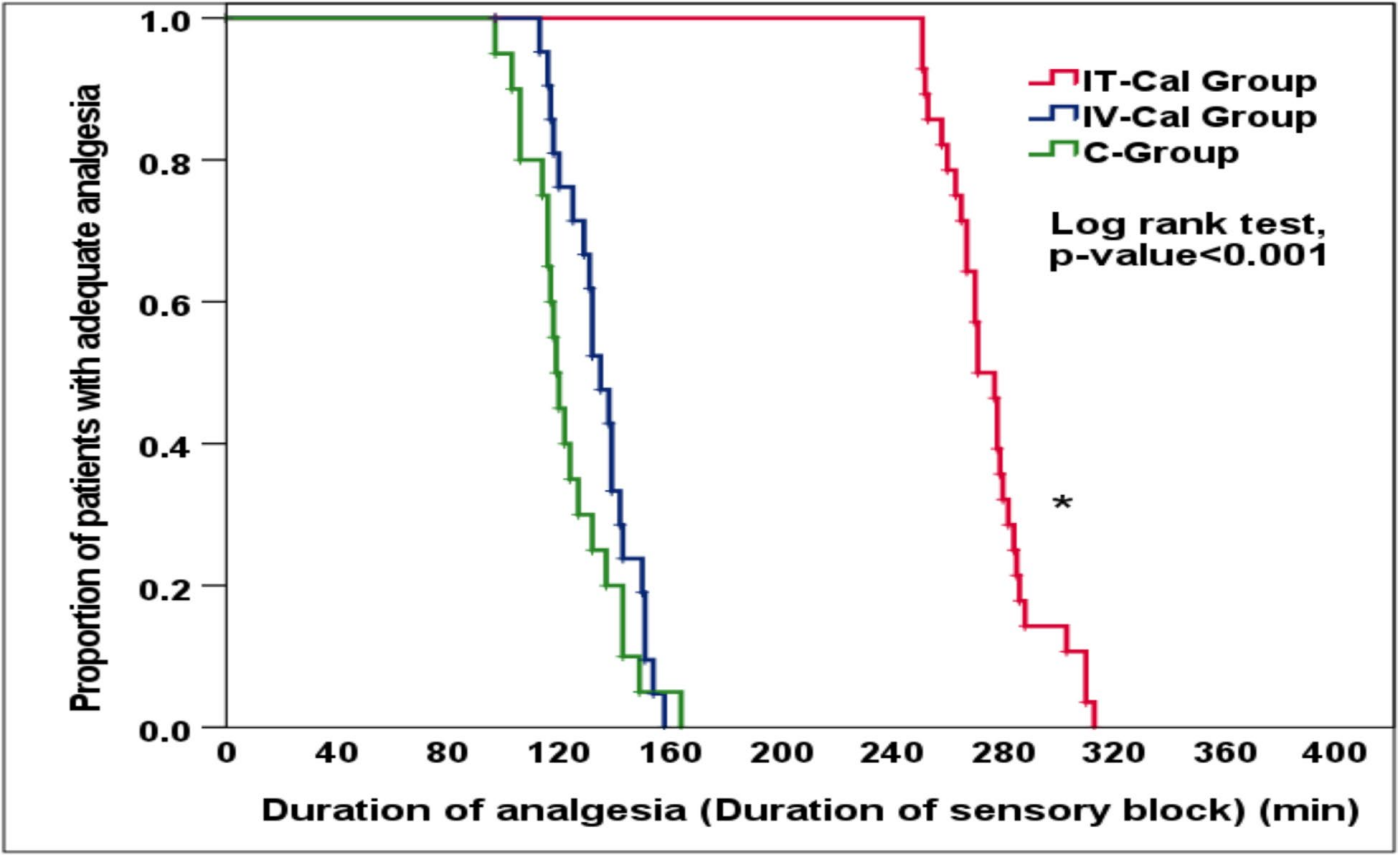
Kaplan–Meier curve for rate of first request of rescue analgesia between study groups. (* Significant)

One hundred twenty-four patients were screened and ninety patients were randomized (Fig. 2). Demographic characteristics were comparable between groups while the incidence of failed SA was significantly lowest in the IT-CAL Group (*P* = 0.018) (Table 1). Among the cases with successful SA, the onset of sensory block, the duration of sensory block (duration of analgesia) (Fig. 3), the duration of motor block and time to two-segment regression were longer in the IT-CAL Group among the three groups (*P* < 0.001) (Table 1).

**Table 1:** Demographic factors, characteristics of spinal anesthesia and intraoperative vital parameters between study groups

Variables		IT-Cal Group (*n* = 29)	IV-Cal Group (*n* = 29)	C-Group (*n* = 29)	*p*-value
**Age; (years)**		35.6 ± 3.6	34.4 ± 5.7	32.9 ± 5.5	*0.123
**Gender**	**Male**	29 (100.0%)	29 (100.0%)	29 (100.0%)	NA
**BMI; (kg/m**^**2)**^		28.8 ± 2.0	29.5 ± 2.4	28.5 ± 2.6	*0.265
**Failed Spinal Anesthesia; (n, %)**		1 (3.4%) a	8 (27.6%) b	9 (31.0%) b	**†0.018§**
		**IT-Cal Group (*n* = 28)**	**IV-Cal Group** **(*n* = 21)**	**C-Group (*n* = 20)**	
**Sensory Block (SB)**					
• **Onset of SB; (min)**		2.5 ± 0.7 a	3.5 ± 1.1 b	3.8 ± 1.6 b	* < 0.001§
• **Time to the highest SB level; (min)**		7.9 ± 1.2 a	17.1 ± 4.2 b	19.4 ± 4.5 b	* < 0.001§
• **Maximum SB level; T4, T5, T6**		15/11/2	9/10/2	4/10/6	**‡**0.094
• **Time to two-segments regression; (min)**		161.8 ± 5.9 a	101.1 ± 4.5 b	97.7 ± 7.5 b	* < 0.001§
• **Duration of sensory block; (min)**		275.8 ± 17.6 a	134.9 ± 13.5 b	123.7 ± 16.8 b	* < 0.001§
**Motor Block (MB)**					
• **Onset of MB; (min)**		4.7 ± 0.8 a	5.4 ± 0.9 b	5.9 ± 1.2 b	* < 0.001§
• **Time to reach complete MB; (min)**		11.0 ± 2 a	16.6 ± 2.2 b	18.1 ± 2.8 b	* < 0.001§
• **Duration of MB; (min)**		153.4 ± 18.4 a	123.0 ± 13.9 b	111.9 ± 16 b	* < 0.001§
**Duration of surgery; (min)**		70.1 ± 4.1	71.7 ± 4.1	72.0 ± 2.5	*0.184
**Intraoperative fluid intake; (ml)**		897.1 ± 56.4	919.4 ± 44.7	921.5 ± 35.6	*0.143

Data are presented as Mean ± SD or number & (%). *BMI* Body Mass Index, *NA* Not applicable. *ANOVA, **†**Chi square test, **‡**Fisher’s Exact test. Homogenous groups had the same symbol "a or b" based on post hoc Bonferroni test. **§**Significant

**Fig. 4:**
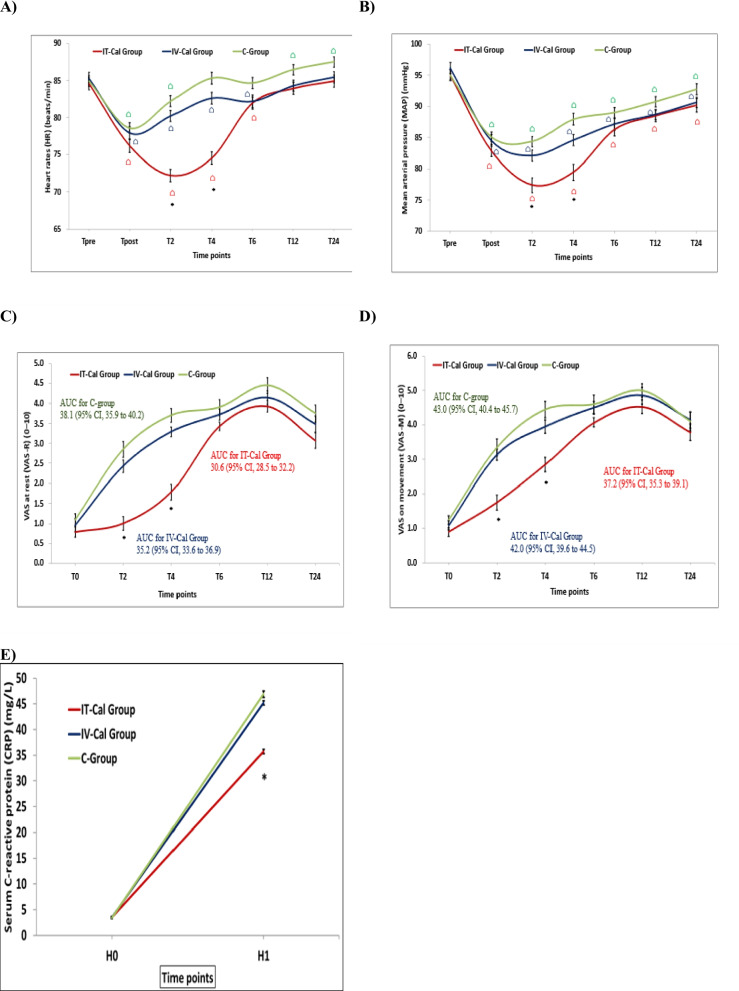
Hemodynamic variables changes, pain scores and surgery-related stress response between study groups;** A** Heart rate changes, **B** Mean arterial pressure changes, **C** Visual analog scale (VAS) scores at rest (VAS-R); Mean ± SD of initial (VAS-R) values during request for rescue analgesia in IT-Cal Group, IV-Cal Group and C-Group were 4.3 ± 0.5, 6.2 ± 1.0 and 6.6 ± 1.4 respectively. It was significantly lowest in IT-Cal Group (*P* < 0.001) with no significant differences between the other two groups (*P* > 0.050). **D** VAS on movement (VAS-M) and **E** Serum C-reactive protein (CRP) (mg/L). Tpre; Before spinal anesthesia (SA), Tpost; 20 min after SA, T0; 30 min postoperative, T2; 2 h postoperatively, T4; 4 h postoperatively, T6; 6 h postoperatively, T12; 12 h postoperatively, T24; 24 h postoperatively, H0: Baseline (1h before the operation) and H1: 24 h after the operation. (*Significantly different group based on post hoc Bonferroni test following ANOVA test. Times significantly different from Tpre had the symbol “⌂” based on post hoc Dunnett`s test following RMANOVA test)

The research team reported that IT-Cal Group had the lowest HR and MAP at 2 h and 4 h after surgery compared to the other two groups (*P* < 0.001) (Fig. 4). The HR and MAP measurements (from Tpost till T4) were less than Tpre measurements in all study groups (*P* < 0.001) (Fig. 4).

**Fig. 5:**
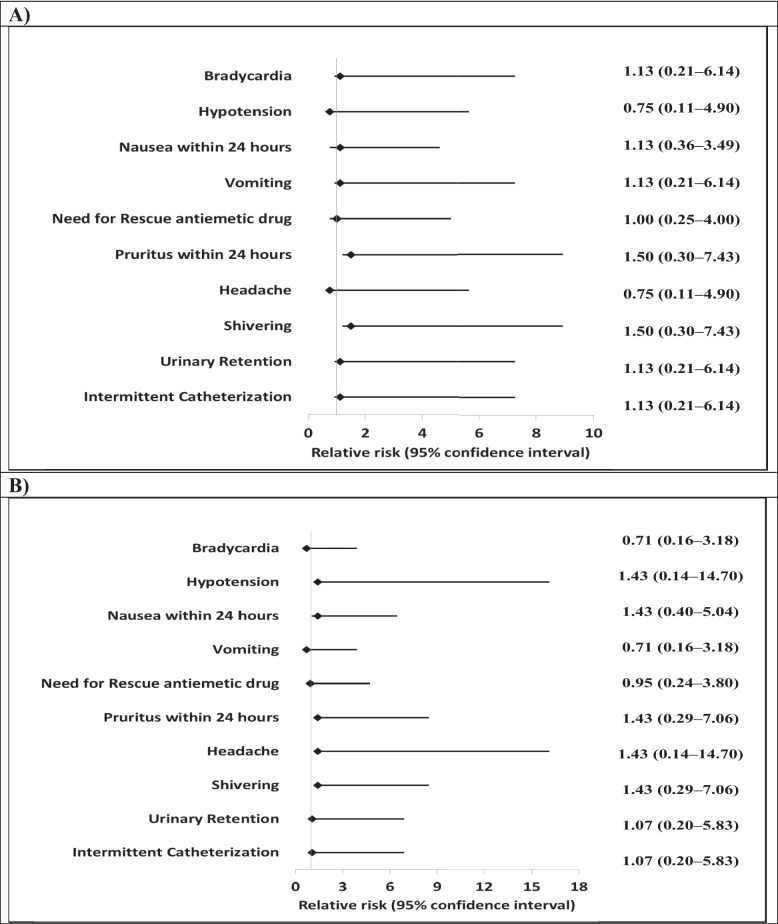
Relative risk of side effects **A** IT-Cal Group relative to IV-Cal Group. **B** IT-Cal Group relative to C- Group

Patients in IT-Cal Group showed the lowest PO pain scores at rest and on movement in comparison to the other two groups at 2 h, 4 h, area under the curve (AUC) of the pain scores perceived over 24 h after surgery, and the initial VAS-R during request for rescue analgesia (*P* < 0.001) (Fig. 4) and the research team found no differences between IV-Cal & C-Groups (*P* > 0*.*05) (Fig. 4). In addition, patients of IT-Cal Group reported lower serum CRP levels 24 h after surgery in comparison to the other two groups (*P* < 0.001) (Fig. 4) with no differences between IV-Cal & C-Groups (*P* > 0*.*05) (Fig. 4). Correspondingly, the IT-CAL Group patients had the longest time to first request for analgesia, the lowest loading, maintenance and total morphine consumption as well as the shortest time to first ambulation in addition to the highest patients’ satisfaction grades 24 h after surgery (Table 2). Regarding side effects, IT-Cal Group was not different when compared to IV-Cal Group nor to C-Group (Table 2, Fig. 5).

**Table 2:** Clinical outcomes and side effects between study groups

Variables			IT-Cal Group (*n* = 28)	IV-Cal Group (*n* = 21)	C-Group (*n* = 20)	*p*-value
**Clinical Outcomes:**						
• **Length of PACU stay; min**			26.6 ± 2.2	27.4 ± 2.3	28.2 ± 2.5	*0.072
• **Time to first request for analgesia; min**			275.8 ± 17.6 a	134.9 ± 13.5 b	123.7 ± 16.8 b	* < 0.001§
• **Loading morphine consumption; mg**			8.4 ± 1.3 a	10.1 ± 1.6 b	10.8 ± 1.4 b	* < 0.001§
• **Maintenance morphine Consumption; mg**			10.8 ± 1.7 a	14.7 ± 2.2 b	16.0 ± 1.9 b	* < 0.001§
• **Total Morphine Consumption; mg/24h**			19.1 ± 2.9 a	24.8 ± 3.8 b	26.8 ± 3.2 b	* < 0.001§
• **Time to first ambulation (min)**			382.7 ± 7.4 a	817.5 ± 6.9 b	822.8 ± 9 b	* < 0.001§
**Side Effects; n, %**						
• **Bradycardia**			3 (10.7%)	2 (9.5%)	3 (15.0%)	**‡**0.803
• **Hypotension**			2 (7.1%)	2 (9.5%)	1 (5.0%)	**‡**0.999
• **Nausea within 24 h**			6 (21.4%)	4 (19.0%)	3 (15.0%)	**‡**0.926
• **Vomiting**			3 (10.7%)	2 (9.5%)	3 (15.0%)	**‡**0.803
• **Need for Rescue antiemetic drug**			4 (14.3%)	3 (14.3%)	3 (15.0%)	**‡**0.999
• **Pruritus within 24 h**			4 (14.3%)	2 (9.5%)	2 (10.0%)	**‡**0.901
• **Need for Rescue antipruritic treatment**			0 (0.0%)	0 (0.0%)	0 (0.0%)	NA
• **Degree of sedation 24 h after surgery**		• **Alert, oriented**	26 (92.9%)	17 (81.0%)	16 (80.0%)	**‡**0.393
		• **Drowsy, oriented**	2 (7.1%)	4 (19.0%)	4 (20.0%)	
• **Median SpO2 (%)**			98.3 ± 0.7	97.9 ± 0.8	97.7 ± 0.9	*0.058
• **Respiratory Depression**			0 (0.0%)	0 (0.0%)	0 (0.0%)	NA
• **Headache**			2 (7.1%)	2 (9.5%)	1 (5.0%)	**‡**0.999
• **Shivering**			4 (14.3%)	2 (9.5%)	2 (10.0%)	**‡**0.901
• **Urinary Retention**			3 (10.7%)	2 (9.5%)	2 (10.0%)	**‡**0.999
• **Intermittent Catheterization**			3 (10.7%)	2 (9.5%)	2 (10.0%)	**‡**0.999
**Hospital length of stay (LOS); h**			35.7 ± 1.7	36.2 ± 2.2	37.1 ± 2.0	*0.062
**Patients’ Satisfaction Grades 24 h after surgery**	**Dissatisfied**		2 (7.1%)a	8 (38.1%)b	9 (45.0%)b	**†0.002§**
	**Neutral**		12 (42.9%)a	10 (47.6%)a	9 (45.0%)a	
	**Satisfied**		14 (50.0%)a	3 (14.3%)b	2 (10.0%)b	

Data are presented as Mean ± SD or number & (%). PACU: Post-Anesthesia Care Unit. NA: Not applicable. *ANOVA, **†** Chi square test, **‡** Fisher’s Exact test. Homogenous groups had the same symbol "a or b" based on post hoc Bonferroni test. **§**Significant

## Discussion

This study demonstrated that intrathecally administered calcitonin decreased the incidence of failed SA, created a favorable PO analgesia and decreased the PO CRP levels in tramadol-abuse patients undergoing unilateral open inguinal hernia repair.

Acute PO pain with insufficient pain management will result in persistent postsurgical pain, which has an incidence of 30–50%. Through interdisciplinary cooperation, multimodal analgesia has been included into more successful PO rehabilitation routes in conjunction with nonopioid analgesics and/or regional methods, such as ERAS and “fast tracks” [30]. In spite of effective PO pain relief provided by intrathecal opioids, multiple side effects including PONV, pruritus and RD may occur particularly at higher doses [31].

Substance abuse is an evolving problem in our community [32]. The present study included males only because of fears of social stigma that come with substance use disorders (SUDs) and cultural barriers in Egypt [7]. People, especially those in their working years, use tramadol to try to boost their work power and performance, which may be the reason for the rise in tramadol dependence given Egypt's challenging economic conditions, which have recently forced people to work long hours in physically demanding jobs [7]. Given to legal restrictions and personality traits, patients may have concerns to acknowledge their addiction problems, an issue that is a great obstacle to researchers in Egypt [6, 7]. It was observed that failed SA is frequently encountered in such patients, and tramadol-abuse might be accused as a cause [3].

The incidence of failed SA ranges from 0.9% to 25.3%, indicating heterogeneity in causes and outcomes. Many alleged causes were proposed for failed SA which may include – but not restricted to—pseudo-successful lumbar puncture, anatomical difficulties, lack of experience, inadequate concentration, inadequate volume and/or resistance to local anesthetics [33]. However, drug addiction, was not raised as one of these causes [3]. A possible explanation of failed SA is that tolerance can occur to local anesthetic receptors with the tolerance of opioid receptors [3, 5], and this is reflected clinically as delay in the onset, decrease in the duration or even complete failure of SA [3]. The research team considered calcitonin as an additive to local anesthetic other than opioids to ameliorate the action of intrathecally injected local anesthetic (Fig. 1).

Calcitonin is a polypeptide hormone targeting primally calcium homeostasis [11]. Different clinical trials and more than 20-year market experience confirmed its safety profile [34]. Calcitonin anti-nociceptive properties have been concluded by different studies in a variety of painful neuropathic conditions, migraine [35] or acute PO pain [36, 37]. The Investigators theorized that its potential analgesic properties are created by mechanisms unrelated to its osteoclastic inhibitory effect (Fig. 1, 14–19). Despite the promising conclusions of its analgesic efficacy, wide range of safety and limited side-effects, salmon calcitonin has been infrequently studied in acute pain management [38].

In accordance to our results Pravin et al., advocated the use of calcitonin as an intrathecal adjuvant in patients undergoing SA for infraumbilical operations due to increasing the duration of PO analgesia in a dose-dependent proportion [37]. Similarly, a previous study concluded that 100 IU IT-CAL received with 3 ml 0.5% heavy bupivacaine provided the longest duration of analgesia compared with the other 2 groups [39]. Congruent with our findings, Miralles et al., reported that 100 IU IT-CAL was an effective analgesic with minimal side effects when combined with subarachnoid lidocaine 1 mg/kg compared to subarachnoid lidocaine plus saline [40]. Also, Gabopoulou et al., proved that 100 IU IT-CAL was a suitable alternative for fentanyl to control acute PO pain when combined with bupivacaine 0.5% as analgesic epidural mixture with stable hemodynamic results, and eliminating PO hyperglycemia in patients undergoing total hip arthroplasty [36].

It is worth saying that in the current study, the perception of pain in patients of IT-CAL Group was not only delayed but also showed lower initial VAS-R scores than the other 2 groups. Despite the fact that the rebound pain was observed in peripheral regional blocks [41], the lower initial VAS-R scores recorded in patients of IT-CAL Group highlighted the necessity to study the phenomenon of rebound pain in SA. As an adjunct to the hyperbaric bupivacaine in SA, the research team observed that 100 IU IT-CAL not only extended PO analgesia but also made a gradual cessation of the sensory block giving the PO pain management team a chance to start an optimal analgesic regimen to prevent PO perception of severe pain.

The analgesic potentials of salmon calcitonin were observed when used for the treatment of different types and causes of pain. A previous study reported that a strong analgesic effect was noticed in terminal cancer patients with unbearable pain who received 100 IU IT-CAL with no important side-effects developed [42]. In addition, Jaeger and Maier documented that early administration of IV infusion of salmon calcitonin was an efficient treatment for phantom limb pain after amputation [38]. Also, Elsheikh and Amr found that adding 50 IU calcitonin to epidural steroid and local anesthetic injection could be considered a new therapeutic modality for pain relief of lumbar spinal stenosis [43].

The current study demonstrated absence of a meaningful effect of intravenously administered calcitonin in either decreasing the incidence of failed SA in tramadol-abuse patients nor improving the block characteristics of SA. Since the exact analgesic mechanism of calcitonin is unclear, the research team raises questions about the neuraxial calcitonin administration rendering analgesia locally or through systemic absorption? Many clinical trials confirmed the analgesic effectiveness of subcutaneous [38], intravenous or intranasal calcitonin [44], in different acute or chronic pain situations [11]. On contrary to the other studies [11], the absence of analgesic effect of IV-CAL in this study might be attributed to the fact that the research team used only one single dose while all other studies used it systemically for many days or even weeks to show its beneficial effects [11].

The results of the current study were in accordance with other clinical trials and by the 20 years post-marketing experience [11, 34, 38] which confirmed that salmon calcitonin is safe and the frequency of side effects was comparable between calcitonin and control groups (Table 2, Fig. 5).

Despite the fact that tramadol is still mostly unregulated in the developing countries, the prevalence of abuse and knowledge of the dangers have been rising [45]. Tramadol abuse possesses detrimental influences on the central nervous system. It induces a state of oxidative stress and alters monoamine neurotransmission in rats. It also induces a state of maladaptive plasticity in brain structures [46]. On the other hand, a previous study reported that intrathecal porcine calcitonin stimulates the release of Met5-enkephalin-like peptides from the rat spinal cord [47] which could explain the beneficial role of IT-Cal in tramadol-abuse patients in this study.

Given to opioid tolerance associated with history of a substance use disorder [3], additional considerations should be directed our focus towards identifying these patients prior to surgery, evaluating pain more objectively, and using multimodal analgesia in conjunction with nonopioid analgesics and/or regional methods to reduce tolerance and avoid withdrawal symptoms [30, 31]. In a multimodal analgesic regimen, nonopioids minimize the negative effects of opioids while providing additive or synergistic analgesia through distinct mechanisms. Early mobilization, early enteral feeding, and mitigation of the PO stress response are the main tenets of a multimodal approach [30, 31].

### Research merits

The current study had many merits; first, it provided an alternative safe non-narcotic adjuvant to boost the local anesthetic in SA specially in cases whom failed SA will be expected [3, 4]. Second, comparing IT-CAL to IV-CAL gave a clue for the optimal choice of drug administration route needed to get the most of salmon calcitonin analgesic effect. Third, the research team highlighted the need for adjuvants to avoid the rebound pain specially in substance abuse patients. Fourth, using morphine titration protocol, allowed giving small boluses of morphine that consequently decreased the risk of adverse events that associate morphine accumulation despite increasing the time for pain relief. Fifth, using area under the curve of the pain scores which represents the percentage of perceived pain over the whole study period rather than the individual time points that might be fluctuating in intensity.

### Limitations

This study had limitation; first; it was performed in a single center study while the nature of the included patients demands a nationwide multi-center study to raise the problem of tramadol-abuse in our community and also to recommend different solutions to the problem of failed SA in such patients. Second; the dose of IV-calcitonin used in this study could have created a significant effect if the dose or frequency was increased.

Third; despite using the DSM-5 criteria for SUDs yet, building the history of SUDs based on the patient`s self‑reporting only represented a potential limitation of this study and the research team recommends that confirming the SUDs reporting by relatives in future studies could overcome this limitation.

## Conclusions

One hundred IU intrathecal calcitonin administration as an adjuvant to hyperbaric bupivacaine was associated with lower total morphine consumption, attenuated stress response and lower incidence of failed spinal anesthesia in tramadol abuse patients undergoing unilateral open inguinal hernia repair when compared with intravenous calcitonin.

## Supplementary Information

Supplementary Material 1.

## Data Availability

The datasets supporting the conclusions of this study are available from the corresponding author.

## Abbreviations

AUC: Area under the curve,
Cal: Calcitonin
CRP: C-reactive protein
IT: Intrathecal
IV: Intravenous
NSS: Normal saline solution
PONV: Postoperative nausea and vomiting
RD: Respiratory depression
SUDs: Substance use disorders
