# Supplementary material for: Efficacy of coadministration of calcitonin and hyperbaric bupivacaine in spinal anesthesia for unilateral open inguinal hernia repair in tramadol abuse patients: a randomized controlled trial
Source: BMC Anesthesiol. 2026 Mar 28;26:208. doi: 10.1186/s12871-026-03736-9 (PMC13045071; doi:10.1186/s12871-026-03736-9)
Supplement: Supplementary file 1 — Supplementary Material 1. [file 12871_2026_3736_MOESM1_ESM.docx]

**Laboratory Analysis**

**- Urine Sample:** Fresh urine specimens were collected in sterile well capped cups under supervision of trustful personnel. Urine can be processed immediately after centrifugation at 1500 xg or stored at 2-8 ˚C and assay within 7 days after collection, or at -20˚C for 6 months. Repeated freezing and thawing should be avoided. The recommended pH range for urine specimens is 4.0-10.0. The clinical pathology specialist obtained another sample for testing if adulteration of the sample was suspected.

- **Blood Sample:** Two milliliters (2 mL) of venous blood were withdrawn under complete aseptic conditions from each subject included in the study one hour preoperatively (H0) and 24 hours postoperatively (H1)**.** The blood was left to clot for 30 minutes in sterile dry vacutainers. The serum was then separated by centrifugation at 1000 xg for 15 minutes for the immediate assessment of CRP. Hemolyzed samples were discarded.

**Analytical Methods:**

**a) Tramadol assay:**

1- **Urine Samples** were tested using Rapid Drugs of Abuse (7tests) Panel supplies by Hanza Medic (852 small industry zone-industrial zone north Katameya Ain Sokhna road-3rd settlement-New Cairo-Cairo) for the qualitative assessment of drugs and/or their metabolites in human urine.

2- **Positive results** will be confirmed by **chromatographic method** using DANI Master gas chromatography with a flame ionization detector (FID) supplied by DANI Instruments SpA Plot No. Pap R-91, Ttc Industrial Area, Rabale ,Navi Mumbai400701 Maharashtra, India). Cut off value of tramadol: 102 ng/mL Cut off value of tramadol metabolites (O-DSMT): 95 ng/mL.

**Reference:** Dawling, S (2011): Gas Chromatography. In: Clarke’s Analysis of Drugs and Poisons. 4^th^ edition. Moffat, A C ; Osselton, M D and Widdop, B eds Pharmaceutical Press 1 Lambeth High Street, London SE1 7JN, UK 1559 St Paul Avenue, Gurnee, IL 60031, USA.pp:636-717.

**b) C reactive protein assay:**

The analysis was done using immunoturbidimetric method on Cobas c 501 autoanalyzer supplied by Roche Diagnostics (GmbH D-68298 Mannheim Germany)**,** Reference intervals: < 5 mg/L.

**Reference:** Burtis CA, Ashwood ER, eds. Tietz Fundamentals of Clinical Chemistry, 5th ed. Pa: WB Saunders Co 2001;332-333.
